# SUPPORT Tools for evidence-informed health Policymaking (STP) 7: Finding systematic reviews

**DOI:** 10.1186/1478-4505-7-S1-S7

**Published:** 2009-12-16

**Authors:** John N Lavis, Andrew D Oxman, Jeremy Grimshaw, Marit Johansen, Jennifer A Boyko, Simon Lewin, Atle Fretheim

**Affiliations:** 1 Centre for Health Economics and Policy Analysis, Department of Clinical Epidemiology and Biostatistics, and Department of Political Science, McMaster University, 1200 Main St. West, HSC-2D3, Hamilton, ON, Canada, L8N 3Z5; 2 Norwegian Knowledge Centre for the Health Services, P.O. Box 7004, St. Olavs plass, N-0130 Oslo, Norway; 3 Clinical Epidemiology Program, Ottawa Health Research Institute, Administration Building, Room 2-017, 1053 Carling Ave., Ottawa, ON, Canada, K1Y 4E9; 4 Norwegian Knowledge Centre for the Health Services, P.O. Box 7004, St. Olavs plass, N-0130 Oslo, Norway; 5 Health Research Methodology Programme, 1200 Main St. West, HSC-2D1, Hamilton, ON, Canada, L8N 3Z5; 6 Norwegian Knowledge Centre for the Health Services, P.O. Box 7004, St. Olavs plass, N-0130, Oslo, Norway; Health Systems Research Unit, Medical Research Council of South Africa; 7 Norwegian Knowledge Centre for the Health Services, P.O. Box 7004, St. Olavs plass, N-0130, Oslo, Norway; Section for International Health, Institute of General Practice and Community Medicine, Faculty of Medicine, University of Oslo, Norway

## Abstract

*This article is part of a series written for people responsible for making decisions about health policies and programmes and for those who support these decision makers*.

Systematic reviews are increasingly seen as a key source of information in policymaking, particularly in terms of assisting with descriptions of the impacts of options. Relative to single studies they offer a number of advantages related to understanding impacts and are also seen as a key source of information for clarifying problems and providing complementary perspectives on options. Systematic reviews can be undertaken to place problems in comparative perspective and to describe the likely harms of an option. They also assist with understanding the meanings that individuals or groups attach to a problem, how and why options work, and stakeholder views and experiences related to particular options. A number of constraints have hindered the wider use of systematic reviews in policymaking. These include a lack of awareness of their value and a mismatch between the terms employed by policymakers, when attempting to retrieve systematic reviews, and the terms used by the original authors of those reviews. Mismatches between the types of information that policymakers are seeking, and the way in which authors fail to highlight (or make obvious) such information within systematic reviews have also proved problematic. In this article, we suggest three questions that can be used to guide those searching for systematic reviews, particularly reviews about the impacts of options being considered. These are: 1. Is a systematic review really what is needed? 2. What databases and search strategies can be used to find relevant systematic reviews? 3. What alternatives are available when no relevant review can be found?

## About STP

*This article is part of a series written for people responsible for making decisions about health policies and programmes and for those who support these decision makers. The series is intended to help such people ensure that their decisions are well-informed by the best available research evidence. The SUPPORT tools and the ways in which they can be used are described in more detail in the Introduction to this series *[1]. *A glossary for the entire series is attached to each article (see Additional File *1*). Links to Spanish, Portuguese, French and Chinese translations of this series can be found on the SUPPORT website *http://www.support-collaboration.org/. *Feedback about how to improve the tools in this series is welcome and should be sent to: *STP@nokc.no.

## Scenarios

*Scenario 1: You are a senior civil servant and will be submitting a brief report to the Minister regarding evidence about a high-priority problem, options to address the problem, and implementation considerations. You are concerned about whether the current draft of the report profiles research evidence that has been synthesised in a systematic and transparent way. You want to ensure that your staff have found the most relevant systematic reviews in the limited time available to them*.

*Scenario 2: You work in the Ministry of Health and have been given a few hours to prepare a brief report about a problem, options to address it, and implementation considerations. All that you have been told is that the report should draw on any relevant systematic reviews that can be found within this time frame*.

*Scenario 3: You work in an independent unit that supports the Ministry of Health in its use of evidence in policymaking. You are preparing a detailed research report for the Ministry of Health about what is known and not known about a problem, options to address it, and implementation considerations. You have been told to find all relevant systematic reviews and you have been given two weeks to do this, but you want guidance on how to do this in a thorough and efficient way*.

## Background

**Figure 1:**
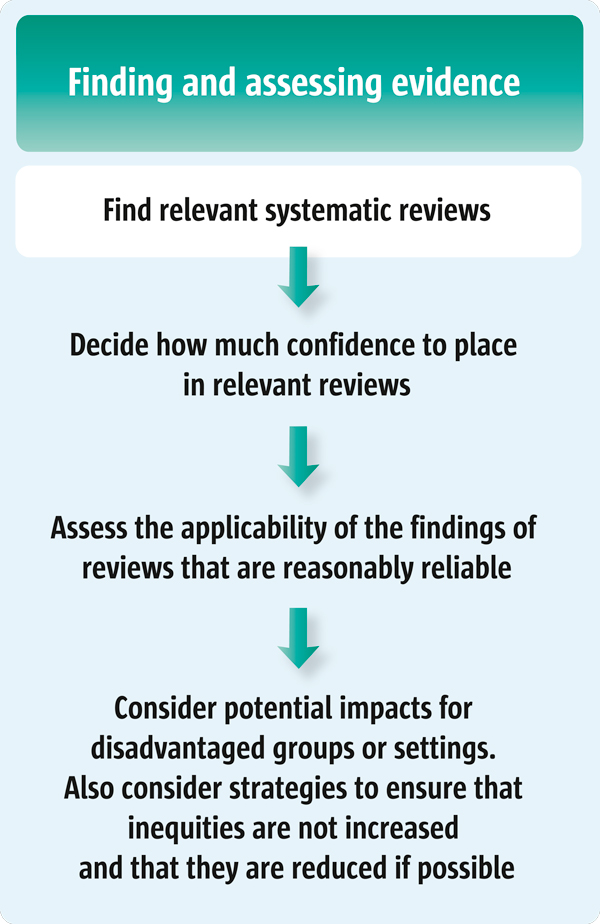
**Finding and assessing systematic reviews to inform policymaking**.

This article suggests a number of questions that policymakers (Scenario 1) might ask their staff to consider when preparing a brief report regarding the evidence about a high-priority problem, options to address the problem, and implementation considerations. For those who support policymakers (Scenarios 2 and 3), this article suggests a number of questions to guide the search for systematic reviews, particularly reviews about the impacts of options being considered. This article is the first of four articles in this series about finding and assessing systematic reviews to inform policymaking (see also Articles 8-10 [2-4]). Figure 1 outlines the steps involved in finding and assessing systematic reviews to inform policymaking.

Systematic reviews are increasingly seen as a key source of information for policymaking, particularly in assisting with framing options and describing their impacts [5]. Systematic reviews offer four key advantages over single studies in characterising the impacts of an option:

1. They reduce the likelihood that policymakers will be misled by research (by being more systematic and transparent in the identification, selection, appraisal and synthesis of studies)

2. They increase confidence among policymakers about what can be expected from an option (by increasing the number of units for study)

3. They allow policymakers to focus on *assessing *the applicability of the findings of systematic reviews to their own setting (instead of also having to find and synthesise the available research evidence on their own). The reviews also allow policymakers to focus on collecting and synthesising other types of evidence, such as local evidence about technical feasibility, the fit with dominant values and the current provincial/national mood, and the acceptability of potential options in terms of budget workability and their likely degree of political support or opposition, and

4. They allow stakeholders, including public interest groups and civil society groups to contest research evidence constructively because it is arranged in the reviews in a more systematic and transparent way [5,6]

The first two advantages listed above - namely, the reduction of bias and increase in precision (to use the terminology of researchers) - apply only to systematic reviews of impacts, some of which will include the statistical synthesis of findings as a final step. In these instances, the reviews are referred to as *meta-analyses *[7].

Although not the principal focus of this article, systematic reviews are also increasingly used as key sources of information in the clarification of problems and providing complementary perspectives on options. Systematic reviews can also be conducted for:

• Administrative database studies and community surveys that help to place problems in comparative perspective

• Observational studies that help to describe the likely harms of an option, and

• Qualitative studies that help to understand the meanings that individuals or groups attach to a problem, how and why options work, and stakeholder views about (and experiences with) particular options

These issues are discussed further in Article 4 in this series (which focuses on the process of clarifying a problem) and Article 5 (which focuses on framing options to address a problem) [8,9]. There are many methodological approaches that can be used in systematic reviews of qualitative research evidence (or in systematic reviews of both qualitative and quantitative research evidence within the same review) including a narrative summary, thematic analysis, grounded theory, meta-ethnography, a meta-study, realist synthesis, cross-case techniques, content analysis, a case survey, qualitative comparative analysis, and Bayesian meta-analysis [10,11].

Several constraints have hindered the wider use of systematic reviews in policymaking. The first key constraint is the limited awareness of their value. Policymakers require synthesised research evidence and systematic reviews are able to provide this in a way that is both systematic and transparent. Many policymakers and researchers with influence in health systems initially believed that systematic reviews could only include randomised controlled trials and required some form of statistical synthesis [12]. For them, the value of these reviews lay only in assessing the effectiveness of healthcare interventions. This belief was underpinned by misconceptions. A second key constraint relates to the retrievability of systematic reviews. Policymakers need timely access to relevant high-quality systematic reviews that are retrievable using the terminology of policymakers. A systematic review of the factors that influence the use of research evidence in policymaking found that timing/timeliness increased the likelihood of research being used by policymakers [6,13]. In the past, policymakers have not been able to search databases using terms familiar to them [14] but this, as we discuss below, has now changed.

A third key constraint relates to the degree to which systematic reviews can be easily understood and interpreted. Policymakers need access to user-friendly summaries of systematic reviews that are written in ways that highlight what they need to know to clarify a problem or describe the costs and consequences of options to address the problem. In the past, even if searches were successful, they may have retrieved structured abstracts and full reviews that had been written in a way that failed to highlight the types of information that policymakers were seeking [14]. Again, as we explain below, this situation has also changed.

## Questions to consider

The following questions can guide policymakers in the process of finding systematic reviews to inform policymaking:

1. Is a systematic review really what is needed?

2. What databases and search strategies can be used to find a relevant systematic review?

3. What alternatives are available when no relevant review can be found?

### 1. Is a systematic review really what is needed?

Before conducting a search for systematic reviews it is first necessary to confirm whether a systematic review is really what is needed. Systematic reviews may be appropriate if, for example, a policy question that is posed addresses a specific health system arrangement or a specific programme, service or drug. They may also be useful for specific implementation strategies that target consumers (e.g. citizens and healthcare recipients) or healthcare providers (with or without some specification of the people, comparisons and outcomes of interest). Article 5 in this series addresses how to structure questions related to the impacts of options [8].

But an overview of systematic reviews could provide helpful information if the question at hand relates to a broad category (or several broad categories) of health system arrangements, programmes, services or drugs, or implementation strategies. A particular overview of systematic reviews, for example, was found to be helpful by many policymakers because it examined the impacts of a full array of options that could be used to improve the supply, distribution, efficient use and performance of healthcare providers [15]. A policy brief that draws on a range of systematic reviews could also prove to be helpful. This would be the case if the question posed by policymakers addresses a spectrum of concerns ranging from the clarification of a problem, the framing of options and the description of their costs and consequences, through to key implementation considerations. The Program in Policy Decision-Making/Canadian Cochrane Network and Centre (PPD/CCNC) database described below could prove helpful in finding both overviews of systematic reviews and policy briefs, as well as systematic reviews. Policy briefs are described in further detail in Article 13 in this series [16].

Systematic reviews are likely to be *unhelpful *if a question pertains to local evidence, such as local evidence about on-the-ground realities and constraints, the values and beliefs of citizens, interest group power dynamics, institutional constraints, and donor funding flows. Article 11 addresses considerations related to finding and using local evidence to inform policymaking [17].

### 2. What databases and search strategies can be used to find a relevant systematic review?

When it has been decided that a systematic review is needed, and when the question that the review needs to address relates to the impacts of (or more generally what is known about) health system arrangements, the PPD/CCNC database can be prioritised as a search tool. This is because it is accessible without charge, it has a particular focus on health system arrangements, and it provides links to user-friendly summaries (and, in their absence, scientific abstracts) (see Table 1 for a description of this and other databases). The database captures both systematic reviews that address questions about impacts *and *systematic reviews that address other types of questions.

**Table 1:** Databases to search for systematic reviews

Database	Comments
PPD/CCNC database	**Features**
	• Accessible online at no cost
	• Focused exclusively on governance, financial and delivery arrangements within health systems
	• Contains Cochrane reviews of impacts, other reviews of impacts, and reviews that address other types of questions (e.g. reviews of qualitative studies), as well as overviews of systematic reviews and policy briefs
	• Provides links to user-friendly summaries (when they exist) and to scientific abstracts
	**What is in it?**
	• Systematic reviews that address any type of question about governance, financial and delivery arrangements within health systems
	• Overviews that identify and synthesise the many systematic reviews that address a specific health systems issue or challenge
	**How can it be searched?**
	• Type of governance, financial and delivery arrangement (by clicking on the relevant category)
	• Type of systematic review, namely review of impacts, Cochrane review of impacts, and review addressing another type of question
	• Type of overview, namely policy brief written primarily for policymakers and overview of systematic reviews written primarily for researchers
	**What resources are provided for search results?**
	• Link(s) to a user-friendly summary that highlights decision-relevant information (if available)
	- Australasian Cochrane Centre (ACC) Policy Liaison Initiative (primarily for policymakers in Australia)
	- Database of Abstracts of Reviews of Effects (DARE) (primarily for healthcare providers but no limitations per se)
	- Effective Health Care Research Programme Consortium (primarily for healthcare providers and policymakers in low- and middle-income countries)
	- Health-evidence.ca (primarily for public health practitioners and policymakers)
	- Reproductive Health Library (primarily for reproductive health practitioners and policymakers)
	- Rx for Change (primarily for policymakers interested in influencing prescribing behaviour or healthcare provider behaviour more generally)
	- SUPPORT (primarily for policymakers in low- and middle-income countries)
	• Link(s) to a scientific abstract (when available)
	• Link(s) to the full text (which may require a subscription or an access fee)

Cochrane Library	**Features**
	• Online version (without full-text reviews) accessible at no cost
	• Contains health-focused Cochrane reviews of impacts (Cochrane Database of Systematic Reviews) and other reviews of impacts (Database of Abstracts of Reviews of Effects and Health Technology Assessment Database)
	• Cochrane Database of Systematic Reviews provides access to scientific abstracts and user-friendly summaries (targeted at lay people). DARE provides links to user-friendly summaries, and the Health Technology Assessment Database provides access to structured scientific abstracts
	**What is in it?**
	• Systematic reviews that address questions about the impacts of clinical, health service/system and public/population health interventions, as well as health technology assessments (many of which will contain a systematic review)
	**How can it be searched?**
	• Search the entire Cochrane Library or (separately) one of its three most relevant constituent databases
	- Cochrane Database of Systematic Reviews (systematic reviews of impacts produced by members of the Cochrane Collaboration according to defined standards)
	- DARE (systematic reviews of impacts with no restriction on who produced them): Note that the most up-to-date version of this database can be searched separately and that most reviews have a user-friendly summary prepared by the Centre for Reviews and Dissemination
	- Health Technology Assessment Database (health technology assessments, which may contain a systematic review): Note that the most up-to-date version of this database can be searched separately and that most reviews have a summary of the HTA's objective prepared by the Centre for Reviews and Dissemination and a link to the full text (which typically does not require a subscription or access fee)
	**What resources are provided for search results?**
	• A user-friendly summary that highlights decision-relevant information for all reviews in DARE (with some time delay depending on staff workload)
	• A lay summary for all Cochrane reviews
	• A scientific abstract for all Cochrane reviews
	• Link(s) to the full text for all Cochrane reviews (requires a subscription or access fee)

PubMed/MEDLINE	**Features**
	• Accessible online at no cost
	• Contains many types of health-focused studies, not just systematic reviews. A hedge is available to find systematic reviews (including Cochrane reviews)
	• Contains only peer-reviewed articles (i.e. no grey literature)
	• Provides links to scientific abstracts only
	**What is in it?**
	• Both studies and systematic reviews that address any type of question that may be addressed in the biomedical, clinical, health service/system and public/population health literature
	**How can it be searched?**
	• Combine content terms AND terms that will yield systematic reviews, with the terms selected here designed to balance the sensitivity and specificity of a search (emphasising specificity over sensitivity) [19]
	- Cochrane Database Syst Rev [TA] OR search [Title/Abstract] OR meta-analysis [Publication Type] OR MEDLINE [Title/Abstract] OR (systematic [Title/Abstract] AND review [Title/Abstract])
	• Possibly also combine with terms that will identify systematic reviews and studies focused on particular jurisdictions or regions (e.g. low- and middle-income countries) - See Additional File 3
	**What resources are provided for search results?**
	• A scientific abstract (if available)
	• Link(s) to the full text (which may require a subscription or an access fee)
	**Notes**
	• There are versions of MEDLINE that require a subscription (e.g. OVID/MEDLINE)
	• PubMed contains many types of health-focused studies, not just studies of impacts, and hedges are available for many types of studies

However, if the question that a review should answer relates to the description of the impacts of programmes, services or drugs, or of implementation strategies targeting consumers and healthcare providers, then policymakers can access two databases used more commonly by healthcare providers. (The 'Resources' section later in this paper provides links to the databases mentioned.) The Cochrane Library - and specifically the Cochrane Database of Systematic Reviews and the Database of Reviews of Effects contained within it - only captures systematic reviews that address questions about impacts (see Table 1). PubMed captures systematic reviews that address many types of questions. *Hedges *(i.e. validated search strategies) are available to assist with finding systematic reviews in PubMed. Hedges are also used to find systematic reviews in three other databases: CINAHL, EMBASE, and PsycINFO (see Additional File 2 later in this article).

Two additional points are important to consider. Firstly, within any of the databases, policymakers who are interested in describing impacts but are pressed for time, may want to give priority to reviews produced by the Cochrane Collaboration (otherwise known as Cochrane reviews). These reviews have been found to be of higher quality and are updated more frequently than reviews produced by other groups [18]. Secondly, while health technology assessments (or HTAs) *should *typically include a range of economic, social, ethical and legal considerations, as well as a review of the research evidence about the effectiveness of a technology, some HTA reports contain a systematic review that can be applied in contexts other than the one for which the report was produced.

Table 2 provides an example of how groups of policymakers and those who support them can work together to find reviews to address a high-priority issue.

**Table 2:** Finding reviews to support the widespread use of artemisinin-based combination therapy to treat malaria

Evidence-Informed Policy Network (EVIPNet) teams of both policymakers and researchers from seven African countries wanted to come to grips quickly with several broad categories of health system arrangements that could be used to support the widespread use of artemisinin-based combination therapy (ACT). Their search identified three overviews of systematic reviews. The first overview was still in progress and focused on the impacts of particular governance arrangements related to prescription drugs like ACT [20]. The second overview focused on the impacts of alternative financial arrangements in health systems more generally [21]. And the third completed overview focused on the impacts of alternative human resources for health (HRH) configurations [15]. Their search also identified an overview of systematic reviews of the impacts of implementation strategies targeting healthcare providers [22].
Once they had read the overviews of systematic reviews, the policymaker/researcher teams searched for systematic reviews in domains not covered by the overviews. They found:
1. Two systematic reviews about governance arrangements. One addressed the impacts of consumer involvement in decision making and the second addressed governance arrangements related to the private sector (however, the latter review is not a review of impacts per se)
2. Six systematic reviews of the impacts of specific financial arrangements, including incentives for patients (i.e. conditional cash transfers), incentives for prescribers, physician-remuneration arrangements more generally, contracting with the for-profit sector to improve healthcare delivery, reference pricing and other pricing and purchasing policies, as well as one systematic review about what is known about financial arrangements within the private sector (again, this latter study was not a review of impacts as such), and
3. Five systematic reviews of the impacts of specific HRH configurations, including home-based management, lay health workers, and the expansion of the role of outpatient pharmacists and *either *nurses or nurse practitioners instead of physicians. In addition, one systematic review was found about the activities of medicine sellers and how their practice can be improved (this, too, was not an actual review of impacts)
Given that the WHO malaria treatment guidelines of 2006 were based on a comprehensive search for systematic reviews about the impacts of anti-malarial drugs, the teams were able to restrict their additional searches to the time period that followed. Six systematic reviews about anti-malarial drugs were found (published in either 2006 or 2007) and one systematic review about unit-dose packaged anti-malarial drugs was also found.
The searches undertaken by the teams also allowed them to supplement the overview of systematic reviews of the impacts of implementation strategies with seven additional systematic reviews of the impacts of different strategies for achieving desired outcomes. These outcomes included the dissemination and implementation of guidelines, the implementation of guidelines among allied health professionals specifically, influencing prescribing and dispensing, changing medication use, improving antibiotic prescribing in ambulatory care and in hospitals, and the enhancement of medication adherence. Seven systematic reviews were also found on the impacts of specific strategies for bringing about change, including audit and feedback, computerised support for determining drug dosage, continuing-education meetings, educational outreach visits, local opinion leaders, mass media campaigns, and tailored efforts to identify identified barriers to change.
The teams found no systematic reviews of studies examining the feasibility and acceptability of ACT for the home-based management of malaria. They therefore conducted a search for single studies on this topic. One study was found which was conducted in four African sites and had been published in *Malaria Journal*.

### 3. What alternatives are available when no relevant review can be found?

Despite improvements in the ease with which policymakers can search and find systematic reviews in available databases, there will be occasional instances when no review can be found. If policymakers are able to wait between 6 and 18 months (depending on the complexity of the question being asked) and have the necessary resources, one option could be to commission a systematic review from an experienced research group [14]. If, however, the available timeline is shorter than this or resources are limited, policymakers can search for single studies instead. In doing so they are essentially conducting a review themselves, and the more systematically this is done the better. In such situations, policymakers can take issues related to ensuring the quality of reviews into consideration. A web-based tool to support such 'rapid evidence assessments' is described later in this paper, while a further discussion of the quality of reviews is provided in Article 8 [2].

Particular databases can also be prioritised when looking for single studies. PubMed, which includes over 20 million records, is often a good starting point. When searching PubMed, hedges can be used to restrict searches to the types of studies most relevant to a particular type of question. Hedges are also available for other databases. (Please refer to the 'Resources' section of this paper for a list of links to hedges that are particularly relevant to policymakers.)

Some policymakers will only require this basic level of detail related to finding systematic reviews or single studies if they have access to subscription databases and are able to rely on the expertise of librarians (Please see Additional File 2 for a list of subscription access databases). This may be either within their own organisation or through colleagues in other universities and settings. We have summarised additional details about high-priority databases in which to search for systematic reviews, including their content, how they can be searched, and what information is returned from search results (see Table 1). This is particularly useful for policymakers who want to gain access to additional information in order to establish clear expectations among those who support them, as well as for policymakers and librarians who will be conducting searches on their own.

Two additional points are worth noting. Firstly, there has been a steady growth in the number of groups and organisations providing user-friendly summaries highlighting the decision-relevant information contained in systematic reviews. Such summaries are usually an excellent place for policymakers to start (Article 13 provides additional detail about these summaries [16]). Secondly, terms have been identified for PubMed in order to help with the identification of systematic reviews and studies focused on low- and middle-income countries. This is particularly useful for policymakers based in these countries. (Additional File 3 at the end of this article provides a list of terms that can be used in searches for systematic reviews or studies focused on these countries.)

While many of the prioritised databases above provide free online access, such access often does not include full-text systematic reviews. In such cases, it will be necessary for policymakers and those who support them (and librarians) to make use of the mechanisms that have been created to allow for the free or low-cost retrieval of the full-text systematic reviews they have identified through their database searches (see Table 3 for a list of these mechanisms).

**Table 3:** Mechanisms through which to retrieve full-text systematic reviews free of charge or at little cost once identified through database searches

Mechanism	Comments
**HINARI**	**Who is eligible to use it?**
	• Institutions in selected low- and middle-income countries have either free access or low-cost access. To check if an institution is already registered or if an institution is located in a country that is eligible for free or low-cost access, go to: HINARI
	**How can it be accessed?**
	• An institution must register and all staff are then given unlimited access
	• Alternatively if a computer is recognised as being based in an eligible country, users may access Highwire Free Access for Developing Countries (which includes HINARI and other selected resources)
	**What resources are provided for research results?**
	• A scientific abstract and full-text article for all included journals

**Cochrane Library**	**Who is eligible to use it?**
	• Institutions in selected countries have free access - to check if a country (or region) is covered by a programme for low-income countries or by a subscription, go to: Cochrane Library
	**How can it be accessed?**
	• Country-or region-specific access details are available at the same site
	**What resources are provided for research results?**
	• A scientific abstract, lay summary, and full-text review for all Cochrane reviews, as well a summary of some form for the three most relevant constituent databases described in Table 1
	**Note**
	• The Cochrane Library can also be accessed through HINARI

**Journals**	**Who is eligible to use them?**
	• Anyone
	**How can they be accessed?**
	• Websites of open-access journal publishers
	- BioMed Central (journals beginning with BMC and select others)
	- OpenJournals Publishing (many journals beginning with 'South African' and select others)
	- Public Library of Sciences (journals beginning with PLoS)
	- SciELO (Scientific Electronic Library Online) (many journals from Latin America and the Caribbean)
	• Directories of open-access and/or free journals
	- Directory of Open Access Journals
	- Free Medical Journals
	- Open J-Gate
	• Repositories through which journal publishers make available articles (often after a defined time period)
	- PubMed Central
	- Bioline International (journals from Brazil, Cuba, India, Indonesia, Kenya, South Africa, Uganda, Zimbabwe)
	**What resources are provided for research results?**
	• A scientific abstract and full-text article for all included journals

Three key options are available:

1. The Health Inter Network Access to Research Initiative (HINARI) which provides institutions in low-income countries with free access to many published reviews and studies

2. The Cochrane Library which provides free access to Cochrane reviews in low-income countries and in countries with a national subscription, and

3. Journals that make their content available free of charge either as soon as they are published or after a defined period of time (e.g. one year)

Three additional methods warrant mention. It may be worthwhile identifying the institution where the authors of a review are based in case they have made it available free of charge on their institution's website. It may also be possible to contact the authors directly by email. Finally, Google Scholar may be used to track down a full-text review if the review is in the public domain and the correct citation is known.

## Conclusion

Systematic reviews are increasingly seen as a key source of information to inform policymaking, particularly in assisting with framing options and describing their impacts. They are also used to assist with a range of questions about a problem, options to address the problem, and implementation considerations. The PPD/CCNC database is a good source for finding systematic reviews that address a range of questions about health system arrangements, as well as overviews of systematic reviews and policy briefs. The Cochrane Library (particularly the Cochrane Database of Systematic Reviews and the Database of Reviews of Effects) and PubMed are both good sources of systematic reviews that address questions about the impacts of programmes, services and drugs. When systematic reviews cannot be found and timelines and resources permit, policymakers could commission a systematic review or conduct their own rapid evidence assessment.

## Resources

### Useful documents and further reading

- McKibbon A, Wyer P, Jaeschke R, Hunt D. Finding the evidence. In Guyatt G, Rennie D, Meade MO, Cook DJ (Editors). Users' Guides to the Medical Literature: A Manual for Evidence-Based Clinical Practice. Second Edition. New York: McGraw Hill Medical, 2008; pp. 29-58.

### Links to websites

- Program in Policy Decision-making/Canadian Cochrane Network and Centre (PPD/CCNC) database: http://www.researchtopolicy.ca/search/reviews.aspx - Source of systematic reviews of studies about health system arrangements (benefits, harms, key features, and the views and experiences of stakeholders).

- Cochrane Library's Cochrane Database of Systematic Reviews (CDSR) and Database of Abstracts of Reviews of Effects (DARE): http://www.thecochranelibrary.com and http://www.york.ac.uk/inst/crd/signup_form.htm (to sign up for electronic updates from DARE) - Source of systematic reviews of programmes, services and drugs (including benefits and possibly harms), as well as health technology assessments, which sometimes contain systematic reviews.

- PubMed: http://www.ncbi.nlm.nih.gov/pubmed and http://www.ncbi.nlm.nih.gov/corehtml/query/static/clinical.shtml#reviews (to use the 'hedge' for reviews) - Source of systematic reviews addressing a range of questions, as well as single studies.

- Health Information Research Unit: http://hiru.mcmaster.ca/hiru/hiru_hedges_home.aspx - Source of 'hedges' (i.e. validated search strategies) to find systematic reviews and a variety of types of studies.

- Rapid Evidence Assessment Toolkit: http://www.gsr.gov.uk/professional_guidance/rea_toolkit/index.asp - Web-based toolkit to assist policymakers and those who support them to find and use research evidence as comprehensively as possible within tight time constraints, which includes a summary of the differences between a rapid evidence assessment and a systematic review and when a rapid evidence assessment might be used.

## Competing interests

The authors declare that they have no competing interests.

## Authors' contributions

JNL prepared the first draft of this article. ADO, JG, MJ, JAB, SL and AF contributed to drafting and revising it.

## Acknowledgements

Please see the Introduction to this series for acknowledgements of funders and contributors. In addition, we would like to acknowledge Andrew Booth and Julie Glanville for helpful comments on an earlier version of this Article.

This article has been published as part of *Health Research Policy and Systems *Volume 7 Supplement 1, 2009: SUPPORT Tools for evidence-informed health Policymaking (STP). The full contents of the supplement are available online at http://www.health-policy-systems.com/content/7/S1.

## Supplementary Material

Additional file 1 Glossary Click here for file

Additional file 2 Databases that require subscription access and ideally the support of a librarian Click here for file

Additional file 3 Terms that will identify in Ovid MEDLINE studies that mention low- and middle-income countries Click here for file
