# Supplementary material for: SUPPORT Tools for evidence-informed health Policymaking (STP) 7: Finding systematic reviews
Source: Health Res Policy Syst. 2009 Dec 16;7(Suppl 1):S7. doi: 10.1186/1478-4505-7-S1-S7 (PMC3271834; doi:10.1186/1478-4505-7-S1-S7)
Supplement: Additional file 2 — Databases that require subscription access and ideally the support of a librarian [file 1478-4505-7-S1-S7-S2.doc]

**Additional File 2: Databases that require subscription access and ideally the support of a librarian**

| **Database** | **Comments** |
| --- | --- |
| [CINAHL](http://www.cinahl.com/prodsvcs/cinahldb.htm) | **What is in it?**   - Both systematic reviews and studies that address any type of question (i.e. not just reviews and studies of impacts) that may be covered in the nursing and allied health literature   **How can it be searched?**   - Combine content terms AND terms that will yield systematic reviews, with the terms selected here designed to optimise the sensitivity and specificity of a search [1] - Confidence intervals (in MH Exact Subject Heading) OR ‘dt’ (in Word in Major Subject Heading) OR Systematic review (in PT Publication Type) (in CINAHL provided by EBSCO) - Possibly also combine with terms that will identify systematic reviews and studies focused on particular jurisdictions or regions (e.g. low- and middle-income countries)   **What resources are provided for search results?**   - A scientific abstract (when available) |
| [EMBASE](http://www.ebscohost.com/cinahl/) | **What is in it?**   - Both systematic reviews and studies that address any type of questions that may be covered in the biomedical and clinical literature   **How can it be searched?**   - Combine content terms AND terms that will yield systematic reviews, with the terms selected here designed to optimise the sensitivity and specificity of a search [2] - Meta-analys:.mp. OR search:.tw. OR review.pt. (in EMBASE provided by Ovid) - Possibly combine also with terms that will identify systematic reviews and studies focused on particular jurisdictions or regions (e.g. low- and middle-income countries)   **What resources are provided for search results?**   - A scientific abstract (when available) |
| [PsycINFO](http://www.apa.org/psycinfo/) | **What is in it?**   - Both systematic reviews and studies that address any type of question that may be covered in the psychology literature   **How can it be searched?**   - Combine content terms AND terms that will yield systematic reviews, with the terms selected here designed to optimise the sensitivity and specificity of a search [3] - Control:.tw. OR effectiveness.tw. OR risk:.tw. (in PsycINFO provided by Ovid) - Possibly combine also with terms that will identify systematic reviews and studies focused on particular jurisdictions or regions (e.g. low- and middle-income countries)   **What resources are provided for search results?**   - A scientific abstract (when available) |
| Other databases for which optimal methodology filters for systematic reviews have not yet been developed | **Region-specific interfaces to several of the above-mentioned databases**   - [Virtual Health Library](http://www.virtualhealthlibrary.org/php/index.php?lang=en) (Latin America and Caribbean Region)   **Regional databases**   - [African Index Medicus](http://indexmedicus.afro.who.int/) - [African Journals Online](http://ajol.info/) - [Index Medicus for the WHO Eastern Mediterranean Region](http://www.emro.who.int/his/VHSL/Imemr.htm) - [Index Medicus for South-East Asian Region](http://library.searo.who.int/index.php?option=com_content&view=article&id=48&Itemid=58) - [LILACS](http://bases.bireme.br/cgi-bin/wxislind.exe/iah/online/?IsisScript=iah/iah.xis&base=LILACS&lang=i) (Latin America and Caribbean Region) - [Western Pacific Region Index Medicus](http://wprim.wpro.who.int/SearchBasic.php)   **Global databases with specific disciplinary areas of focus**   - [EconLit](http://www.econlit.org/) (Economics) - [International Bibliography of the Social Sciences](http://www.ovid.com/site/catalog/DataBase/108.jsp) (Social sciences) - [International Political Science Abstracts](http://www.ovid.com/site/catalog/DataBase/110.jsp) (Political science) - [ISI Web of Science](http://apps.isiknowledge.com/WOS_GeneralSearch_input.do?product=WOS&search_mode=GeneralSearch&SID=R25kEckeEfI65NHhjNJ&preferencesSaved=&highlighted_tab=WOS) (Arts and humanities, sciences, and social sciences – citation indices) - [PAIS (Public Affairs Information Service) International](http://www.csa.com/factsheets/pais-set-c.php) (Public affairs) - [Sociological Abstracts](http://www.csa.com/factsheets/socioabs-set-c.php) (Sociology) - [Wilson Business Abstracts](http://library.dialog.com/bluesheets/html/bl0553.html) (Management) - [Worldwide Political Science Abstracts](http://www.csa.com/factsheets/polsci-set-c.php) (Political science)   **Disease/condition databases**   - [TropIKA](http://www.tropika.net/svc/collection/review/) (Tropical diseases) |

**References**

1. Wong SS, Wilczynski NL, Haynes RB: **Optimal CINAHL search strategies for identifying therapy studies and review articles.** *J Nurs Scholarsh* 2006, **38:**194-9.

2. Wilczynski NL, Haynes RB: **EMBASE search strategies achieved high sensitivity and specificity for retrieving methodologically sound systematic reviews.** *J Clin Epidemiol* 2007, **60:**29-33.

3. Eady AM, Wilczynski NL, Haynes RB: **PsycINFO search strategies identified methodologically sound therapy studies and review articles for use by clinicians and researchers.** *J Clin Epidemiol* 2008, **61:**34-40.
