# Supplementary material for: SUPPORT Tools for evidence-informed health Policymaking (STP) 7: Finding systematic reviews
Source: Health Res Policy Syst. 2009 Dec 16;7(Suppl 1):S7. doi: 10.1186/1478-4505-7-S1-S7 (PMC3271834; doi:10.1186/1478-4505-7-S1-S7)
Supplement: Additional file 3 — Terms that will identify in Ovid MEDLINE studies that mention low- and middle-income countries [file 1478-4505-7-S1-S7-S3.doc]

**Additional file 3: Terms that will identify in Ovid MEDLINE systematic reviews and studies that mention low- and middle-income countries**

1. Developing Countries/

2. Medically Underserved Area/

3. Africa/ or "Africa South of the Sahara"/ or Asia/ or South America/ or Latin America/ or Central America/

4. (Africa or Asia or South America or Latin America or Central America).tw.

5. (American Samoa or Argentina or Belize or Botswana or Brazil or Bulgaria or Chile or Comoros or Costa Rica or Croatia or Dominica or Equatorial Guinea or Gabon or Grenada or Hungary or Kazakhstan or Latvia or Lebanon or Libya or Lithuania or Malaysia or Mauritius or Mexico or Micronesia or Montenegro or Oman or Palau or Panama or Poland or Romania or Russia or Seychelles or Slovakia or South Africa or "Saint Kitts and Nevis" or Saint Lucia or "Saint Vincent and the Grenadines" or Turkey or Uruguay or Venezuela or Yugoslavia).mp. or Guinea.tw. or Libia.tw. or libyan.tw. or Mayotte.tw. or Northern Mariana Islands.tw. or Russian Federation.tw. or Samoa.tw. or Serbia.tw. or Slovak Republic.tw. or "St Kitts and Nevis".tw. or St Lucia.tw. or "St Vincent and the Grenadines".tw. [UMIC]

6. (Albania or Algeria or Angola or Armenia or Azerbaijan or Belarus or Bhutan or Bolivia or "Bosnia and Herzegovina" or Cameroon or China or Colombia or Congo or Cuba or Djibouti or Dominican Republic or Ecuador or Egypt or El Salvador or Fiji or "Georgia (Republic)" or Guam or Guatemala or Guyana or Honduras or Indian Ocean Islands or Indonesia or Iran or Iraq or Jamaica or Jordan or Lesotho or "Macedonia (Republic)" or Marshall Islands or Micronesia or Middle East or Moldova or Morocco or Namibia or Nicaragua or Paraguay or Peru or Philippines or Samoa or Sri Lanka or Suriname or Swaziland or Syria or Thailand or Tonga or Tunisia or Turkmenistan or Ukraine or Vanuatu).mp. or Bosnia.tw. or Cape Verde.tw. or Gaza.tw. or Georgia.tw. or Kiribati.tw. or Macedonia.tw. or Maldives.tw. or Marshall Islands.tw. or Palestine.tw. or Syrian Arab Republic.tw. or West Bank.tw. [LMIC]

7. (Afghanistan or Bangladesh or Benin or Burkina Faso or Burundi or Cambodia or Central African Republic or Chad or Comoros or "Democratic Republic of the Congo" or Cote d'Ivoire or Eritrea or Ethiopia or Gambia or Ghana or Guinea or Guinea-Bissau or Haiti or India or Kenya or Korea or Kyrgyzstan or Laos or Liberia or Madagascar or Malawi or Mali or Mauritania or Melanesia or Mongolia or Mozambique or Myanmar or Nepal or Niger or Nigeria or Pakistan or Papua New Guinea or Rwanda or Senegal or Sierra Leone or Somalia or Sudan or Tajikistan or Tanzania or East Timor or Togo or Uganda or Uzbekistan or Vietnam or Yemen or Zambia or Zimbabwe).mp. or Burma.tw. or Congo.tw. or Kyrgyz.tw. or Lao.tw. or North Korea.tw. or Salomon Islands.tw. or Sao Tome.tw. or Timor.tw. or Viet Nam.tw. [LIC]

8. ((rural or remote or nonmetropolitan or underserved or under served or deprived or shortage) adj (communit$ or count$ or area? or region? or province? or district?)).tw.

9. ((developing or less$ developed or third world or under developed or poor$) adj (communit$ or count$ or district? or state? or province? or jurisdiction? or nation? or region? or area? or territor$)).tw.

10. ((middle income or low income or underserved or shortage) adj (communit$ or count$ or district? or state? or province? or jurisdiction? or nation? or region? or area? or territor$)).tw.

11. (lmic or lmics).tw.

12. or/1-11

Note:

1.The filter is based on the World Bank country list of upper-middle-income economies (UMIC), lower-middle-income economies (LMIC), and low-income economies (LIC), which is available at the following URL: <http://web.worldbank.org/WBSITE/EXTERNAL/DATASTATISTICS/0,,contentMDK:20421402~pagePK:64133150~piPK:64133175~theSitePK:239419,00.html>.
